# Radioactive contamination and climate warming affect physiological performance of Chornobyl barn swallows

**DOI:** 10.1371/journal.pone.0329769

**Published:** 2025-08-06

**Authors:** Zbyszek Boratyński, Timothy A. Mousseau, Anders Pape Møller

**Affiliations:** 1 BIOPOLIS, CIBIO/InBio, Research Centre in Biodiversity and Genetic Resources, University of Porto, Campus de Vairão, Portugal; 2 Department of Biological Sciences, University of South Carolina, Columbia, South Carolina, United States of America; 3 Ministry of Education Key Laboratory for Biodiversity Science and Ecological Engineering, College of Life Sciences, Beijing Normal University, Beijing, China; 4 Ecologie Systematique Evolution, Universite Paris-Saclay, CNRS, AgroParisTech, Paris, France; Universiti Teknologi Malaysia, MALAYSIA

## Abstract

Global warming and degradation of natural habitats are the two main factors causing ecophysiological stress on individuals and risk for biodiversity. Hyperthermia is a common response to stress in homeothermic animals, in particular to heat, pathogens and environmental contamination. Resilience of biological systems to global warming may be deteriorated in polluted habitats. Here we investigated how body temperature of a wild bird, the barn swallow (*Hirundo rustica*), responded to global warming while simultaneously exposed to radioactive contamination from the Chernobyl accident. Our results showed that both high air temperatures (t = 15.55, df = 335, p < 0.0001) and elevated environmental radioactive contamination (t = 5.18, df = 8.09, p = 0.0008) increased internal body temperature of individuals. The additive effect suggests that birds might suffer hyperthermia in locally contaminated habitat (1.47% body temperature increase) while simultaneously exposed to globally rising temperatures (1.95% body temperature increase), potentially reducing the fitness of individual and the maintenance of breeding colonies. The cumulative and interactive negative effects of multiple stressors, such as those emerging from increasing habitat degradation and climate change, will likely contribute to biodiversity losses globally.

## Introduction

Global warming is progressing rapidly, affecting processes at different levels of biological organization [1]. Predicting responses to rising temperatures is essential for determining risks for biodiversity. Resilience of biological processes to shifting climate is hampered by increasing deterioration of natural habitats [2]. Cumulative effects of global warming, habitat degradation, and contamination can be a negative force for animal populations [3]. Understanding ecological and physiological mechanisms behind these changes is an essential first step in designing mitigation strategies to safeguard biodiversity and ecosystem services.

Climate change, and rising air temperature in particular, affect animal physiological performance [4]. High environmental temperatures can impact animal water balance and degrade thermoregulation, leading to dehydration and hyperthermia. In extreme situations loss in physiological performance due to exposure to heat can lead to fatalities [5]. Evidence of increased mortality in wildlife, domestic animals and humans with rising air temperatures has accumulated during recent decades [5,6]. Research in the tropics has shown dramatic increases in frequency of massive die-offs during heatwaves for birds and bats [7]. But physiological performance is also impaired at higher latitudes, e.g., temperate birds showed similar thermoregulatory responses as tropical birds, even when exposed to milder temperatures from global warming [8,9]. Likewise, acute and chronic heat reduce livestock productivity and quality [10,11], and increases the risk of mortality in human populations exposed to increasing global temperatures [5], thus indirectly and directly hampering our health and well-being.

Hyperthermia is a common response to heat exposure in animals, especially in passerine birds [12]. Facultative hyperthermia, the elevation of body temperature above normothermic levels, can help to minimize water loss during heat exposure by reducing thermal gradients and allowing non-evaporative heat loss [12]. This non-pathological facultative up-regulation of body temperature represents a temporal physiological increase in set-point of body temperature regulation [13]. Fever, in contrary, is a pathogenic responses to infection with selection benefits [14,15]. Fever can be harmful, e.g., for nervous system [16], when it coincides with other causes of body temperature up-regulation, by increasing risk of acute hyperthermia, such as when febrile animals are exposed to increased air temperatures [3,16]. Although some bird species can tolerate body temperatures as high as 46°C (in particular, passerines from humid and hot areas; [17]), above that threshold heat injury across tissues and organs may cause death. Even small changes in body temperature can affect physiological homeostatic regulation [18]. In such circumstances individuals may suffer fitness loss due to mild stress revealing energetic allocation and trade-offs between investments into reproduction and thermoregulation, e.g., during development of eggs or chicks in birds [19,20].

Physiological responses to pollution, such as to the radioactive contamination from nuclear accidents, waste, mining and processing of radioactive ores, and from medical procedures, may alter organismal responses to other stressors and pathogens [21,22]. Importantly, negative effects of exposure to increasing contamination of natural habitats may impair animal resilience to changing climate and global warming [3]. In response to ionizing radiation, and other stressors affecting DNA and cellular constituents, production of tissue-oriented signals initiate immune and febrile responses, resulting in up-regulation of body temperature [16,21]. Wild barn swallows (*Hirudo* *rustica*) exposed to increased levels of ionizing radiation from the Chernobyl accident showing increased body temperature, when compared to birds from uncontaminated habitats [23]. Exact mechanisms of this observation are unknown, but may involve heat generation and dissipation, as well as energetics of mobility and maintenance processes, potentially leading to overheating and fitness loss when exposed to rising air temperatures. In this study we investigate how barn swallow physiological performance is affected by the interplay between habitat contamination from the Chernobyl accident and global warming. We analyzed a large data set of wild animal core body temperatures collected for birds nesting around Chornobyl City and nuclear accident sites in Ukraine and Belarus. We tested if simultaneous exposure to elevated radioactive contamination and rising environmental temperatures impaired animals performance.

## Materials and methods

### Study animals and area

**Fig 1:**
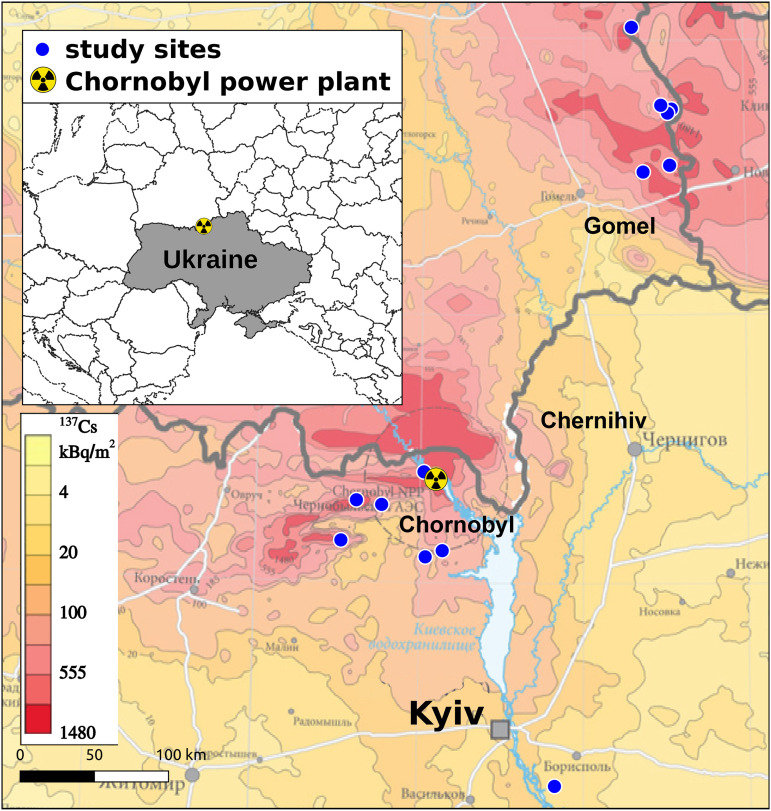
Radioactive contamination around Chornobyl (Ukraine). Study area and environmental contamination in northern Ukraine and south-eastern Belarus, including sites around Chornobyl and Kyiv cities (Ukraine). Radiation background map is modified from DeCort et al 1998 [27], under a CC BY license, with permission from Marc DeCort of the European Commission, original copyright 2015.

Barn swallows (*Hirundo rustica* Linnaeus, 1758) have become a model organism for eco-evolutionary research of responses to radioactive contamination around Chornobyl City, Ukraine [23–26]. Ukrainian barn swallows often nest in abandoned farm buildings (with no livestock), from spring to summer, and migrate for wintering in sub-Saharan Africa. Here we included data collected for 1091 individuals, captured between 25th May and 9th June 2008−2019, including 12 breeding seasons from 13 study sites in Ukraine and Belarus (Fig 1) [23]. Birds were captured at study sites with mist nets, individually marked with bird rings at first capture and immediately phenotyped and released back to the capture area. Individuals of this species showed considerable philopatry, i.e., when found at a given site, they rarely moved to another location in the Chornobyl region. Generally, the maximum recorded breeding dispersal in other European populations was 750 m [28]. The study sites represent a gradient of radiation levels derived from radioactive nucleotides released from Chernobyl nuclear accident in 1984 [27]. Many of the study sites still have high levels of radioactive contamination (Fig 1). Access to study sites and handling of birds followed international regulations, ethical approval by the University of South Carolina Institutional Animal Care and Use Committee (assurance number: A3049-01), and permits from the authorities of the Chernobyl Exclusion Zone.

### Bird body temperature

Core body temperature of barn swallows was measured *in situ* (with a precision of 0.1ºC) with a thermometer (TA804C) equipped with a thermocouple, by inserting the tip of the thermocouple probe 1.0 cm into the cloaca of the birds [29]. These measurements, conducted within 60s, provide consistent readings when repeated (intraclass correlation coefficient = 0.74), and provide reliable estimates of animal internal body temperature [29]. Body mass measured with a Pesola spring balance (with a precision of 0.1g) was recorded along with other morphological (including keel, tarsus and bill dimensions), behavioral (including order of capture and tonic immobility) and phenological (day and hour of capture) data [23].

### Environmental radioactive contamination

In order to relate radioactive contamination to animal body temperature we collected ionizing radiation data from the exact sites of capture of barn swallows. The level of environmental radioactive contamination was repeatedly measured at ground level using a hand-held dosimeter (Inspector, SE International, Inc., Summertown, TN, USA; for details see: [30]). Those measures showed high and significant repeatabilities among days, seasons and years (intraclass correlation coefficient > 0.89) when repeating measurements from the same sites [31]. The results also showed a strong correlation (Pearson’s product-moment = 0.70) with a large ^137^Cs deposition dataset from the European Union Joint Research Center Radioactivity Environmental Monitoring project (Fig 1) [23,32]. Using data reported in [32], ^137^Cs activity levels at our study sites within the Chernobyl Exclusion Zone varied from <3 to >6000 kBq/m^2^ and is the primary source for all gamma radiation stemming from the Chernobyl disaster. ^90^Sr is also abundant in the region ranging from about 6–3300 kBq/m^2^ but being a pure beta emitter is only of concern if ingested. ^241^Am, and Pu isotopes (^238^Pu, ^239^Pu, ^240^Pu) can also be found but at much lower levels (^241^Am: 0.3–128 kBq/m^2^; Pu: 0.06–40 kBq/m^2^) and thus are unlikely to contribute significantly to dose rates.

### Environmental weather conditions

To link weather conditions to animal body temperature we collected weather data predictions for the study sites, years and particular days, for minimum, maximum and mean daily air temperature and relative humidity, recorded 2 m above the ground. Additionally, to estimate general trends in those variables over longer periods of time (1981–2022) in the study areas, we collected monthly June values representative for Chornobyl City only. Minimum, maximum and mean values were obtained with native resolution of 0.5 x 0.625 latitude and longitude degrees, from the National Aeronautics and Space Administration (NASA), Langley Research Center (LaRC) Prediction of Worldwide Energy Resource (POWER), a project funded through the NASA Earth Science/Applied Science Program [11]. The data were obtained from the POWER Project’s Hourly 2.0.0 version on 20.10.2022 [33].

### Statistics

To estimate long term trends in weather conditions (1981–2022) around Chornobyl City (Ukraine) we modeled the effect of year on June minimum, maximum and mean air temperature and relative humidity, with linear regression analysis. We tested if the same trends, for the same weather predictors, are present in the shorter weather data set (2008–2019), restricted to our study period but including all study sites. Next, to reduce correlations among the six weather variables, and to reduce the number of predictors in further analyses, we applied rotated (varimax; on standardized and centered variables) principal component analysis, generating two principal components, referring to environmental temperature and relative humidity (in “psych” v. 2.2.9 R package).

To test if environmental predictors, environmental temperature, relative humidity and radioactive contamination, affect bird physiological performance, measured as body temperature, we applied mixed model analysis. Our analysis included random factors of: individual bird ID (to account for repeated measurements on the same individual; 129 birds had two or more measures), study site (to account for pseudo replication of multiple individuals from the same site), and year (to account for correlation among individual differences among years). We tested for differences between males and females (sex as fixed factor) and their differential response to environmental predictors (interactions between sex and weather variables and radioactive contamination), and included body mass as a continuous predictor of body temperature. We investigated nonlinear responses to environmental predictors by modeling their quadratic terms. Additionally, to test how radioactive contamination modulates bird response to temperature and humidity, we tested two-way interactions among continuous predictors of radioactive contamination and weather variables. Body temperature, mass and radioactive contamination were normalized (log transformed), standardized and centered prior to mixed model analysis (in “lme4” v. 1.1.29 and “lmerTest” v. 3.1.3 R packages) [34].

## Results

**Fig 2:**
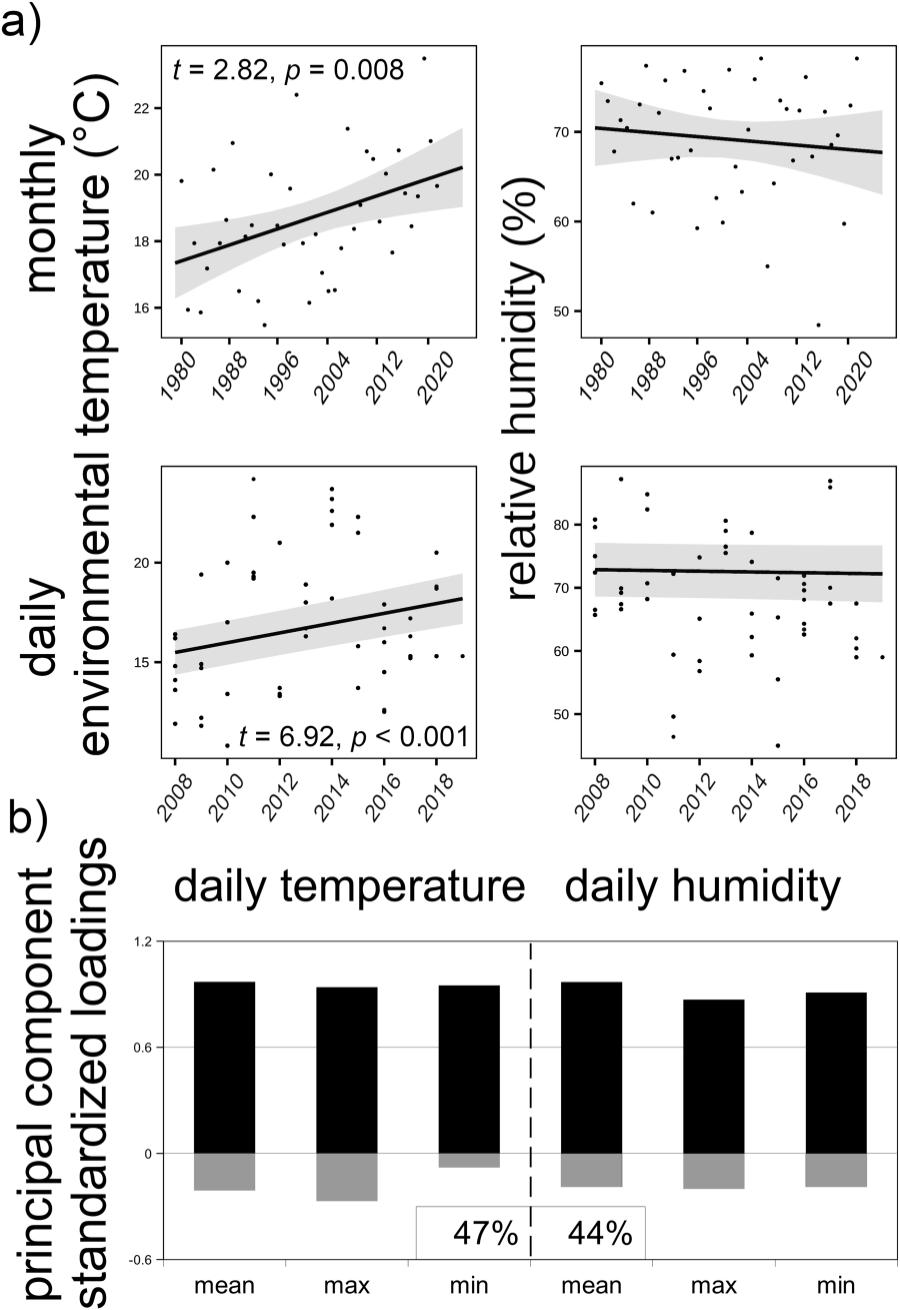
Climatic variation around Chornobyl (Ukraine). a) Trends and variation in climatic conditions around Chornobyl City (upper row) and in exact sampling sites and study years (lower row; from NASA/POWER CERES/MERRA2 projections). Statistics for significant trends in environmental temperatures are presented. b) Standardized loadings (positive – black, negative – gray) for two principal components, from principal component analysis of daily climatic variables (minimum, maximum, mean) for exact study sites, are presented along with percentage of explained variance.

Environmental contamination varied significantly among our study sites in northern Ukraine and south-east Belarus (variance = 3.034 µGy/h), from 0.02 to 8.5 µGy/h, with mean and median of 0.76 and 0.31 µGy/h, respectively (Fig 1). We showed a rise in environmental temperatures, both in the data from our study sites and 2008−2019 years (daily minimum: t = 2.05, p = 0.041, maximum: t = 7.03, p < 0.001, and mean values: t = 6.92, p < 0.001) and in the data from Chornobyl City for 1981−2022 years (monthly June minimum: t = 1.30, p = 0.20, maximum: t = 3.19, p = 0.003, and mean temperatures: t = 2.82, p = 0.008). We did not detect time-trends in relative humidity (Fig 2a). Principal component analysis reduced six correlated weather variables (Pearson’s product moment correlations: −0.45–0.98) to two uncorrelated components, describing 92% of the cumulative variation in minimum, maximum and average daily environmental temperatures and relative humidity (Fig 2b).

Barn swallow body temperature [mean (min-max, variance) = 41.13 (37.3–43.7, 1.21) °C] was weakly but significantly correlated with body mass [mean = 18.81 (15.0–24.0, 2.10) g; r (95% CI) = 0.13 (0.08–0.19), t = 4.71, df = 1244, p < 0.001). The correlation was marginally stronger in males [mean = 18.26 (15.0–21.8, 1.17) g; r (95% CI) = 0.20 (0.13–0.28), t = 4.97, df = 585, p < 0.001] than in females [mean = 19.30 (15.2–24.0, 2.42) g; r (95% CI) = 0.11 (0.04–0.19), t = 2.78, df = 657, p = 0.006]. Body temperature did not differ markedly between males [mean = 41.15 (37.4–43.5, 1.25) °C] and females [mean = 41.11 (37.3–43.7, 1.19) °C; t = 0.58, p = 0.56].

**Fig 3:**
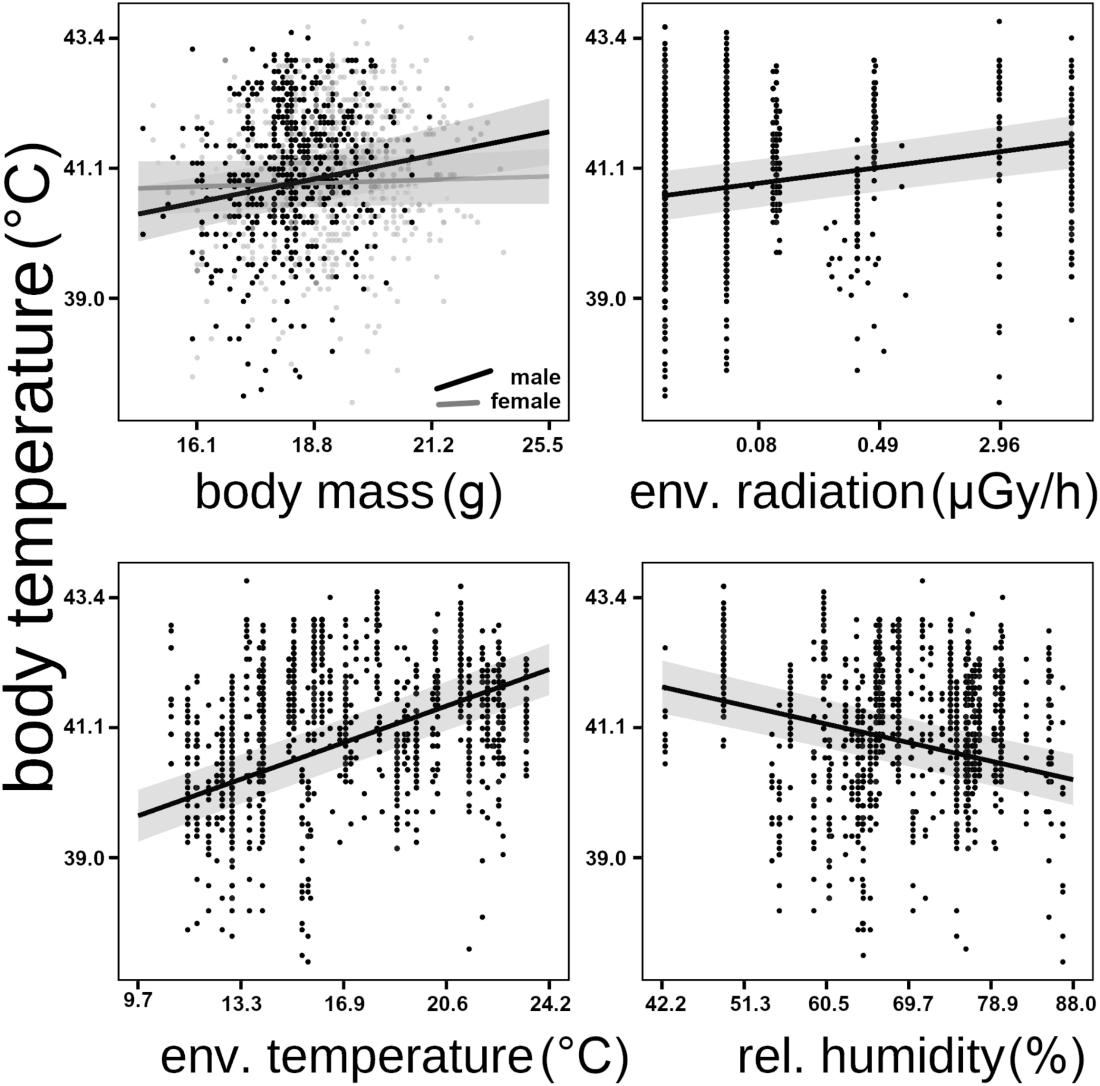
Birds body temperature around Chornobyl (Ukraine). Effects of body mass, environmental radioactive contamination from the Chernobyl accident and weather conditions on body temperature of barn swallows (*Hirundo rustica*). Back transformed predicted values (95%CI) and standardized effects are derived from the statistical model accounting for variation among study sites, years and individuals (Table 1). Mean environmental temperatures and relative humidity are back transformed from principal components (Fig 1; for simplicity only means are presented).

**Fig 4:**
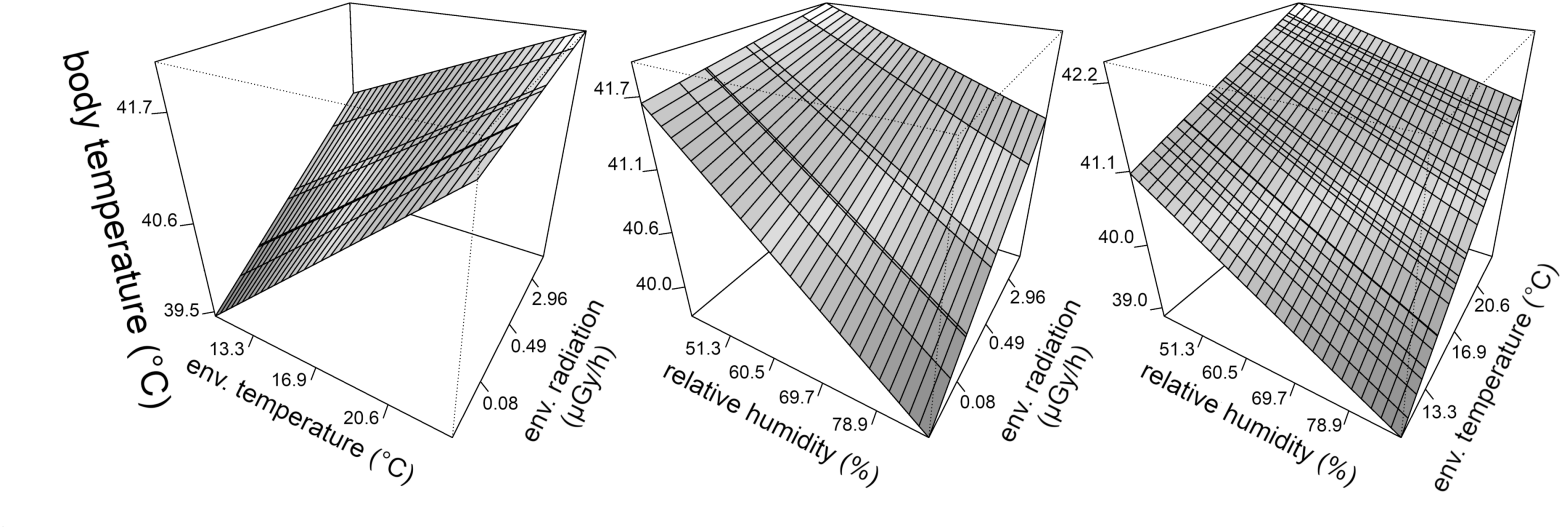
Synergic effects of contamination and climate on body temperature around Chornobyl (Ukraine). Interactive effects of environmental radioactive contamination from the Chernobyl accident and weather conditions on body temperature of barn swallows (*Hirundo rustica*). Back-transformed predicted values and standardized effects are derived from a statistical model accounting for variation among study sites, year and individuals, and estimated interactions. Mean environmental temperatures and relative humidity are back transformed from principal components (Fig 1; for simplicity only means are presented).

Body temperature was higher in barn swallows captured in locations with higher levels of radioactive contamination (Fig 3; Table 1). Body temperature of barn swallows was also higher during days with higher air temperatures, and lower during days with higher relative humidity (with marginal quadratic effect of relative humidity; Table 1). Environmental effects of radioactive contamination and weather conditions were consistent when excluding records for the driest days (S1 File), and when including previously detected individual quality and phenology predictors of body temperature in barn swallows [23]. Additional analyses found significant interactions between radioactive contamination and environmental temperature, radioactive contamination and relative humidity, and between environmental temperature and relative humidity, as predictors of birds body temperature (Fig 4; Table 2).

**Table 1:** Responses of birds body temperature to radioactive contamination and climate around Chornobyl (Ukraine).

	est. (s.e.)	df	t	p
**Intercept**	−0.12 (0.20)	12.35	−0.60	0.56
**sex (male)**	0.07 (0.05)	1006	1.43	0.15
**body mass**	0.04 (0.03)	1208	1.35	0.18
**radioactive cont.**	0.26 (0.05)	8.09	5.18	0.0008
**env. temperature**	0.62 (0.04)	335	15.55	<.0001
**rel. humidity**	−0.34 (0.03)	484	−10.38	<.0001
**rel. humidity^2**	−0.10 (0.02)	607	−4.95	<.0001
body mass * sex	0.14 (0.05)	1207	2.84	0.005

Changes in individually measured absolute (log_10_ transformed) barn swallow (*Hirundo rustica*) body temperature (°C) resulting from environmental radioactive contamination (µGy/h) from the Chernobyl accident, daily environmental temperature (°C) and relative humidity (%), and body mass (g), accounting for variation among individuals, sites and years. Variance (s.d.) of random factors: individual ID = 0.03 (0.17), study site = 0.03 (0.17), study year = 0.38 (0.62) and residual variance = 0.54 (0.73,) for 1246 observations, 1091 individuals, 13 sites and 12 years. Vif < 1.65.

**Table 2:** Interactive responses of birds body temperature to radioactive contamination and climate around Chornobyl (Ukraine).

	est. (s.e.)	df	t	p
**intercept**	−0.14 (0.20)	12.86	−0.69	0.50
**sex (male)**	0.07 (0.05)	1025	1.57	0.12
**body mass**	0.05 (0.03)	1213	1.63	0.10
**radioactive cont.**	0.22 (0.05)	11.73	4.12	0.0015
**env. temperature**	0.60 (0.04)	409	15.00	<.0001
**rel. humidity**	−0.36 (0.04)	289	−9.72	<.0001
**rel. humidity^2**	−0.15 (0.02)	832	−6.54	<.0001
body mass *sex	0.13 (0.05)	1207	2.69	0.007
rad.cont. *env.temp.	−0.13 (0.03)	1115	−4.66	<.0001
rad.cont. *rel.hum.	0.16 (0.03)	1113	4.88	<.0001
env.temp. *rel.hum.	0.07 (0.03)	352	2.03	0.043

Changes in individually measured absolute (log_10_ transformed) barn swallow (*Hirundo rustica*) body temperature (°C) resulting from environmental radioactive contamination (µGy/h) from Chernobyl accident, daily environmental temperature (°C) and relative humidity (%), and body mass (g), accounting for variation among individuals, sites and years. Interactions between climatic conditions and radioactive contamination predicting barn swallows body temperature are presented. Variance (s.d.) of random factors: individual ID = 0.04 (0.19), site = 0.03 (0.17), year = 0.40 (0.63), residual = 0.52 (0.72), for 1246 observations, 1091 individuals, 13 sites and 12 years. Vif < 1.69.

## Discussion

Warming climate and degradation of natural habitats are the two main factors negatively impacting biodiversity globally [35,36]. June air temperatures in Chornobyl City (Ukraine) rose by an average of 1.75°C, from around 18.1 to 19.8°C during the 31 years between 1981–1990 and 2012–2021. Temperatures in our study sites in northern Ukraine and south-eastern Belarus (Fig 1) also rose, by an average of 0.5°C, from around 16.1 to 16.6°C in only seven years (between 2008–2012 and 2015–2019; Fig 2). Data collected during this period from more than 1000 wild barn swallows showed that rising environmental temperatures and contamination from the nuclear accident simultaneously contributed to the elevation of internal body temperature of birds (Figs 3 and 4). Variation in air temperatures between 17–18 and 19–20°C resulted in an increase in body temperature of around 0.8°C (by 1.95%), from 41.0 to 41.8°C. Simultaneously, variation in relative air humidity had limited effect on bird body temperatures. An increase in humidity from 40–60–70–90% coincided with a decrease in body temperature of only 0.1°C (by 0.24%, from 41.5 to 41.4°C). In contrast, even relatively small increases in chronic exposure to ionizing radiation, from 0.02 to 2.9 μGy/h (compared to <0.02 μGy/h prior to the accident, and ≥30 μGy/h in some contaminated locations around Chernobyl [37,38], or to an acute exposure from a single x-ray scans of human chest of 900–3400 μGy/scan) [39], resulted in an increase in body temperature by an average 0.6°C (1.47%), from 40.9 to 41.5°C (Table 1).

The observed levels of air temperatures in our study area (average ~18–20ºC) are below mortality thresholds usually observed, or assumed, for bird species (~46ºC) [4]. Presumably, therefore, barn swallows are still not at risk of mass mortality in Chornobyl, similar to those observed in hotter and more tropical regions and caused by extreme heat waves [7]. Nevertheless, we found here that birds body temperature significantly increased with increasing, over four decades, ambient temperatures (Table 1). This novel results highlight that even mildly rising air temperatures can affect bird thermoregulation [40]. Our individual body temperature data suggested that barn swallows either up-regulate their internal temperatures to diminish water loss [41], or are unable to dissipate heat effectively, thus they suffer overheating when air temperatures rise (Fig 3). Both these non-mutually exclusive mechanisms may pose costs for individuals inhabiting contaminated habitats. Energetic maintenance costs, energy that must be spent for basic life functions by an endothermic animal (i.e., resting/basal metabolic rate), scale positively with animal body temperature [42,43]. Consequently, animals with higher body temperatures, such as birds exposed to radioactive contamination (Table 1), might invest more into foraging to cover likely elevated daily energetic expenses [44]. Altering the level of metabolic costs can have subsequent consequences for fitness and population dynamics in endothermic animals [45,46], such as in the barn swallows studied here. Elevated metabolism may promote overall faster energy turnover the amount of energy to be allocated to performance [47]. But, high maintenance costs may constrain energy allocable to important functions, such as between thermoregulation, mobility and reproduction [48,49], and increase ingestion rate of environmental contaminants. Consequent of contamination, chronic overheating may lead to physiological challenges, such as malfunctions of the neural system (also evident in human [49]) resulting in elevated oxidative stress, depleting fertility [50]. Determining the mechanisms (e.g., oxidative stress, metabolic costs) underlying elevated body temperatures will be important for predicting the sustainability of animal populations in the face of global warming.

The observed rise of barn swallow body temperature across an increasing gradient of radioactive contamination in northern Ukraine and south-eastern Belarus (Table 1) most likely relates to negative effects of ionizing radiation on cellular and physiological homeostasis [18,21]. Around Chornobyl, after the explosion and fire at the nuclear reactor of the power plant in 1986, a large amount of diverse nuclear radioisotopes was released, mainly into the surrounding environment but some were also distributed across the European continent that still persist in the ecosystems [51–53]. The main long-term isotopes of that accident, cesium (^137^Cs), plutonium (^239^Pu) and strontium (^90^S) with half-lives of ~30, ~ 24 000 and ~29 years [54,55], entered the food chain, being ingested with food and water by animals [37]. Consequently, exposure to radionuclides affected animal biology, including reduced reproduction [56,57] and increased frequency of aberrations, such as cataract [30,56], among other effects [25,31,38,56,57]. Exposure to chronic low-dose radioactive contamination may cause febrile responses similar to those observed after intoxication by chemicals [58], or from effects mediated by higher infection rates on host organisms (i.e., immune response). Whichever is the mechanism behind observed body temperature increase (Tables 1 and 2), it poses a risk that animals exposed to high contamination get closer to a thresholds of performance lose, or to the limits of thermoregulation, above which persistence of population is at risk do to fitness decline of individual birds.

Since barn swallows body temperatures in our data increased with both radioactive contamination and rising temperatures (Table 1), as simultaneously showed here for the first time, exposure to increased radioactive contamination may potentially increase animal sensitivity to the negative effects emerging from global warming (Fig 4). However, the effects are less prominent when birds already have high body temperature. Accordingly, in low ambient temperatures, body temperature rises faster due to radiation, than under high ambient temperatures [interactive effect between radioactive contamination and environmental temperature: β = −0.13 (0.03), df = 1115, p < 0.0001; Table 2]. In other words, the relation between body and ambient temperatures is stronger (steeper slope) when animals are exposed to low radioactive contamination, when compared to animals inhabiting high radiation locations (left panel: Fig 4). This interactive result suggest that, while the effects of environmental temperature (t = 15.55, df = 335, p < 0.0001) and radioactive contamination (t = 5.18, df = 8.09, p = 0.0008) are additive (operate in the same direction; Table 1), animals might be reaching their physiological limits (ceiling), or thermoregulation mechanisms may be compromised in highly contaminated habitats. As a result, the normal mechanisms expressed by animals when exposed to increased ambient temperatures can not be applied any more when simultaneously exposed to both environmental stressors (e.g., due to oxidative stress or elevated metabolic costs) [25,59]. Alternatively, animals might be more sensitive to radiation in low ambient temperatures, suggesting that animals foraging more in a cold weather suffer more exposure from radiation ingested with food or due to the increased behavioral activity in contaminated areas. While the first mechanisms could signal synergy of arising risk of radiation and climate change, the second suggests that, to some extend, animals can buffer themselves from such cumulative risk, by reducing activity in hot weather. Even doe the synergy between negative effect of radiation and ambient temperature seems to decrease with increasing levels of both factors (as indicated by negative interaction; Table 2), birds exposed to increased radiation always suffer more from overheating, and perhaps dehydration during warm weather. Therefore, it can be hypothesized that birds simultaneously exposed to increased radiation and climate warming might suffer greater fitness losses, which could at least in part explain the observations that barn swallows frequently abandon their colonies around Chornobyl City [25,38,60,61].

## Conclusions

Our results show that increased radioactive contamination and air temperatures cause up-regulation of birds body temperatures, potentially leading to hyperthermia. While we could not elucidate specific mechanisms responsible for hyperthermia it might have additive effects on animal performance when simultaneously exposed to radiation and warming climate. Damaging effects could arise from increased oxidative stress induced from ionizing radiation and high temperatures. Another possible mechanism includes missed opportunities by birds avoiding activity during warm weather and in contaminated habitats (where additionally birds food abundance might be lower) [62,63]. Growing evidence suggests that behavioral changes, such as decreased activity for energy acquisition needed for reproduction can incur far-reaching consequences for fitness [64]. Avoiding foraging flights, supported by high metabolic work [65] that unavoidably increases physiological heat production, could be a strategy during hot days in already mildly hyperthermic animals in contaminated areas. We hypothesize that habitat degradation and contamination can impair resilience to global change, adding to a growing number of negative consequences of environmental change, such as seasonality mismatching in bird phenology [66]. The correlative nature of our study limits inferring mechanisms, to be approached in future experiments (e.g., in controlled exposure studies). Envisioning appropriate mitigation actions (i.e., restitution of habitats and its mosaic, buffering heat waves) will be essential for safeguarding wildlife exposed to multiple environmental stressors, coincidentally safeguarding human well-being as well [67].

## Supporting information

S1 File Responses of birds body temperature to radioactive contamination and climate around Chornobyl, excluding records with low daily relative humidity. Barn swallow (*Hirundo rustica*) body temperature as predicted by body mass, environmental radioactive contamination from Chernobyl accident, daily environmental temperature and relative humidity, while accounting for variation among individuals, sites and years.
(PDF)

S2 File Inclusivity-in-global-research-questionnaire. (PDF)
