# Supplementary material for: Radioactive contamination and climate warming affect physiological performance of Chornobyl barn swallows
Source: PLoS One. 2025 Aug 6;20(8):e0329769. doi: 10.1371/journal.pone.0329769 (PMC12327629; doi:10.1371/journal.pone.0329769)
Supplement: S1 File — Barn swallow (Hirundo rustica) body temperature as predicted by body mass, environmental radioactive contamination from Chernobyl accident, daily environmental temperature and relative humidity, while accounting for variation among individuals, sites and years. (PDF) [file pone.0329769.s001.pdf]

S1 Table. Responses of birds body temperature to radioactive contamination and climate around Chornobyl.

	est. (s.e.)	*df*	*t*	*p*
intercept	-0.17 (0.21)	13.52	-0.87	0.40
sex (male)	0.06 (0.05)	944	1.25	0.21
body mass	0.05 (0.03)	1138	1.54	0.12
radioactive cont.	0.26 (0.06)	10.87	4.58	0.0008
env. temperature	0.58 (0.04)	498	14.94	<.0001
rel. humidity	-0.28 (0.03)	485	-8.58	<.0001
rel. humidity^2	-0.09 (0.02)	302	-3.78	0.0002
body mass * sex	0.13 (0.05)	1126	2.58	0.010

Barn swallow (*Hirundo rustica*) body temperature as predicted by body mass, environmental radioactive contamination from Chernobyl accident, daily environmental temperature and relative humidity, while accounting for variation among individuals, sites and years, excluding records with low daily relative humidity. Variance (s.d.) of random factors: individual ID = 0.03 (0.17), site = 0.04 (0.20), year = 0.38 (0.62), residual = 0.55 (0.74), for 1170 observations, 1024 individuals, 12 sites and 12 years. Vif < 1.65

S2 Table. Interactive responses of birds body temperature to radioactive contamination and climate around Chornobyl.

	est. (s.e.)	*df*	*t*	*p*
intercept	-0.15 (0.21)	13.90	-0.70	0.49
sex (male)	0.06 (0.05)	972	1.33	0.18
body mass	0.05 (0.03)	1141	1.84	0.066
radioactive cont.	0.22 (0.06)	12.85	3.78	0.002
env. temperature	0.55 (0.04)	530	14.31	<.0001
rel. humidity	-0.29 (0.03)	363	-8.85	<.0001
rel. humidity^2	-0.15 (0.03)	306	-5.76	<.0001
body mass * sex	0.13 (0.05)	1131	2.51	0.012
rad.cont. * env.temp.	-0.12 (0.03)	1075	-4.48	<.0001
rad.cont. * rel.hum.	0.15 (0.03)	1049	5.40	<.0001
env.temp. * rel.hum.	0.02 (0.03)	625	0.57	0.57

Barn swallow (*Hirundo rustica*) body temperature as predicted by environmental radioactive contamination from Chernobyl accident, daily environmental temperature and relative humidity, and body mass, while accounting for variation among individuals, sites and years, excluding records with low daily relative humidity. Interactions between climatic conditions and radioactive contamination predicting barn swallow body temperature are presented. Variance (s.d.) of random factors: individual ID = 0.04 (0.19), site = 0.03 (0.17), year = 0.40 (0.63), residual = 0.52 (0.72), for 1246 observations, 1091 individuals, 13 sites and 12 years. Vif < 1.69.
